# Understanding the paradox between tragedies of the commons and the anticommons: From a cognitive psychology perspective

**DOI:** 10.3389/fpsyg.2022.998642

**Published:** 2022-09-20

**Authors:** Xiaowei Yang, Shiqi Yang, Yuejin Wang, Shuyu Zhang, Songmei Luo

**Affiliations:** ^1^Law School, Ningbo University, Ningbo, China; ^2^School of Economics and Management, Huzhou University, Huzhou, China; ^3^Business School, University of Nottingham Ningbo China, Ningbo, China

**Keywords:** cognitive paradox, tragedy of the commons, tragedy of the anticommons, cognitive psychology, bounded rationality, perceived change, mental accounting

## Abstract

The tragedy of the commons refers to the overuse of resources which are rival in consumption but lack excludability and it also refers to rent dissipation. While the tragedy of the anticommons is a tragedy closely connected with underuse of resources that are rival in consumption and with too strong excludability. The prior studies proved that the tragedy of the commons and the tragedy of the anticommons are symmetric from the perspective of pure mathematics, especially the game theory, which was later refuted by behavioral economics experiments. According to them, the tragedy of the anticommons is severer than the tragedy of the commons. The asymmetry of the tragedy of the commons and the tragedy of the anticommons is a paradox by these different research methods. This paradox shows that there are imperfections in the completely rational economic man hypothesis set up by neoclassical economics. As a fundamental theory, the tragedy of the commons is quite influential in many disciplines, such as microeconomics, public sector economics, ecological economics, environmental economics, management, sociology, property law, and political science. And the tragedy of the anticommons theory has also opened its door of both theoretical research and practical implications since its acceptance by Nobel laureate Buchanan, the main founder of public choice school. Only when theoretical issues are thoroughly discussed and made clear enough, can people avoid misunderstanding or misusing the commons theory. Thus, it is necessary to elucidate the paradox between them. Based on Simon’s bounded rationality, Kahneman and Tversky’s prospect theory, value function, Thaler’s mental accounting, endowment effect, and other cognitive psychological tools, this study clearly shows that agents’ decision-making process is not just based on the long-believed marginal benefit and marginal cost analysis advocated by traditional neoclassical economists. Agents’ decision-making is a process in which agents selectively absorb, code the objective marginal revenue and marginal cost, and feed relevant information to their brain. Therefore, what plays a directly decisive role is not the objective marginal revenue and marginal cost *per se*, but the mentally perceived subjective utility of marginal revenue and marginal cost by the human brain. Followed by this research clue, the paradox between the tragedy of the commons and the tragedy of the anticommons is elucidated from the perspective of cognitive psychology.

## Introduction

Hardin (1968) published a manuscript in Science titled “*The Tragedy of The Commons*,” giving birth to the tragedy of the commons theory. The tragedy of the commons refers to the over-exploitation of rivalry-in-use resources and rent dissipation due to the lack of excludability. In Hardin’s (1968) article, the original prototype of the commons was common pastures in medieval Europe. However, its real value lies in its metaphorical nature. Most ecological environment problems, deterioration of species diversity on the earth, and the space trash problem are related to this metaphor. The tragedy of the anticommons theory was first proposed by Heller, one of the preeminent scholars working on private law theory today, in his nearly 70-page long article published in Harvard Law Journal in 1998. It is a later found underuse tragedy because of resources’ too strong excludability resulting from the over-fragmentation of resources or their property rights. So far, these two theories have played an essential role in environmental economics, microeconomics, law and economics, organizational behavior, management, and other disciplines.

Famous economists, such as Nobel laureate Buchanan, accept the tragedy of the anticommons theory and take the lead in demonstrating the symmetry of tragedy of the commons and the tragedy of the anticommons in pure mathematics (game theory). However, the symmetry has been questioned in the field of experimental economics. Vanneste et al.’s research shows that the welfare loss caused by the anticommons tragedy is more serious than that caused by the commons tragedy in behavioral economics experiments. Symmetry in mathematics (game theory) and asymmetry in behavioral economics experiments constitute an obvious paradox. Considering their huge and wide-spread practical policy influences, if the paradox is not made crystal clear, misunderstanding and misleading in the practical policy process can hardly be avoided. Both the tragedy of the commons and the tragedy of the anticommons have much to do with the so called social dilemma. Looking through this perspective, we can then see that there are a large number of studies having repeatedly proved that cooperative behavior in social dilemmas can be explained by factors such as responsibility and ethics (e.g., Fleishman, 1980; Enzle et al., 1992; Kerr, 1992; Van Dijk and Wilke, 1997; De Cremer and Van Lange, 2001; Parks and Rumble, 2001). Specifically, the causal attributions of cooperation or non-cooperation are opposite for commons dilemma and anticommons dilemma, and social norms revealed in commons dilemma and anticommons dilemma are also opposite (Hiel et al., 2008). In other words, what is socially understood as “just” behavior (cooperation) in the game of the commons may be regarded as “unjust” (non-cooperation) in the game of the anticommons, and vice versa. Moreover, ways to supply public goods by avoiding the tragedy of the commons can also be regarded as the collective action issue, of which the key is to solve the free rider problem (Yong and Choy, 2021). Interestingly, it is proved that preschoolers are sensitive to free riding in a public goods game (Vogelsang et al., 2014). Existing researches closely related to this topic are mainly conducted from the perspective of social dilemma, which lay partial foundation for our research, but the paradox between tragedies of the commons and the anticommons has its own particularity. Still, deep reasons for this paradox, especially from the cognitive psychology perspective, are still lack of exploration.

In order to fill this research gap, we elucidate the paradox between tragedies of the commons and the anticommons quantitatively and theoretically from the perspective of cognitive psychology tools such as prospect theory, value function, mental accounting, and the endowment effect, set up by Kahneman, Tversky, and Thaler, and we propose: The direct decisive factor of decision-maker’s behavior is not a simple comparison of objective marginal revenue and marginal cost, but the subjective perception in their brain, that is, the perceived utility of their expected marginal revenue and expected marginal cost. Cognitive psychology tools, such as prospect theory, mental accounting, and heuristics, play an important role in uncovering the “black box” of information acquisition, classification, and coding in decision-making process. And nowadays it is quite common for scholars to illustrate economic, managerial and cultural phenomena from the perspective of cognition, and knowledge acquisition (for example, Chin et al., 2020, 2022; Duan et al., 2021; Huang et al., 2022). Therefore, this study attempts to analyze the paradox of the tragedy of the commons and the tragedy of the anticommons by using cognitive psychology tools set up by the above-mentioned scholars. Not only will this study contribute directly to a clearer understanding of the paradox, but it will also help to broaden the scope of cognitive psychology and better to understand the underlying driving forces of human behavior. And practically, it may reduce the probability of policy makers’ misunderstanding and misuse of the commons theory.

The rest of this manuscript is arranged as follows: The second part introduces the paradox between the tragedy of the commons and the tragedy of the anticommons in detail. The third part summarizes and reviews the development of cognitive psychology related to this manuscript. The fourth part analyzes the paradox from the cognitive psychology perspective, and the fifth part is the conclusion and future research direction.

## Paradox of tragedies between the commons and the anticommons

Considering that tragedy of the commons has become a familiar terminology in the academic community, while the tragedy of the anticommons is relatively not so familiar to readers, this study will briefly describe the tragedy of the commons but introduce the tragedy of the anticommons in detail.

### From the commons tragedy to the anticommons tragedy

Common resources are rivalry in use but lack excludability, which is the root cause of the tragedy of the commons. Rivalry in use is the attribute that the quantity or quality of the resource is decreased by usage or consumption, and then the marginal utility of future users will be diminished. Excludability refers to the ability or degree of ease by which the owner or actual controller can exclude unwelcome infringers from using or approaching the resource. Obviously, rivalry in use is an inherent property of goods, which can hardly be changed by artificial means. In addition, influenced by such metaphors as the tragedy of the commons, people seem to form a stereotype that “all the commons are doomed to be tragic.” Therefore, adding excludability for rivalry resources seems the key to overcoming the tragedy of the commons. But the question is: The more excludability, the higher resource usage efficiency?

From 1990 to 1994, Heller personally visited Moscow as a legal adviser of the World Bank to help rebuild Russia’s private property rights. He witnessed a strange phenomenon: The streets of Moscow were crowded with people shopping in kiosks, while the storefronts were empty. Russia’s then deputy Prime Minister Vladimir Gaidar turned to Heller for reasons and solutions. Bearing Gaidar’s question in mind, Heller published his anticommons tragedy theory in 1998, in one of his representative articles titled “*The Tragedy of the Anticommons: Property in the Transition from Marx to Markets*,” based on Michelman (1982, 2003) and Ellickson (1993). According to this theory, anticommons tragedy refers to the tragedy of over-fragmented resources or their property rights, leading to over-excluding resources and further underusing resources. The tragedy of the anticommons usually does not mean damage or even destruction to resources. Instead, it refers to a situation in which too many property owners shape obstacles for a potential Pareto improvement to maximize the value of resource usage, or even cause scarce resources cannot be used at all. This situation is particularly prominent when the integration of fragmented resources or their property rights is needed under the influence of technological, economic and social changes.

If n fragmented resources or property rights are integrated, the net revenue (1−δ)π = *R*−*C* (i.e., revenue minus cost) will be created. Suppose the *n* agents (they are excluders to be exact) follow homogeneity hypothesis. In that case, it seems that everyone can gain net benefit (1−δ)π/*n*. However, in reality this potential net benefit (1−δ)π is more likely to be substantially eroded by opportunistic behavior (with a discount rate 0≤δ≤1). If *n* is large enough, the individually rational self-interested agents will greatly increase the cost (*C*) of resource integration. Under the condition of constant income (*R*), (1−δ)π approaches zero. That is, the discount rate (δ) approaches 1. This is because, for the agent who is the first one of being integrated, the share of 1/*n* may be acceptable, but after (*n*−1) agents having been integrated, the agent whose resource constitutes the last but meanwhile essential share, then there is a very high probability for him or her to refuse a 1/*n* share, and instead, a share much larger than 1/*n* is highly likely to be required by this agent. This is the case especially only if successful integration of all resources, or pieces of property rights, can the revenue *R* be realized. Otherwise, *R* will be very small or even close to zero. This assumption is generally in line with reality. For example, inventing a new medicine is not feasible without any key pieces of information (patents). Or to build a factory, small plots are useless unless they are integrated together in proper ways. Moreover, even if we do not change the “rational man” hypothesis, the analysis here can still make sense, because such negotiations are not usually a one-time game, but rather a dynamic game played over time. Therefore it cannot be understood in the logic similar to the ultimatum game. Therefore, unless a unanimous consent distribution agreement is reached in advance, no one is willing to be the first one to be integrated. Through the above mechanism, potential Pareto improvement opportunities will be undermined, which is the basic logic of the tragedy of the anticommons.

According to different causes of underuse and inefficiency of resources, the anticommons tragedy can be divided into legal anticommons and spatial anticommons. The legal type focuses on the over-fragmented property rights of resources caused by legal and institutional factors – too many owners have exclusive property rights or even veto rights on a certain resource. And the space type refers to over-fragmentation on the resource *per se*. Even though the property rights of every tiny piece of the resource are not fragmented, those tiny pieces of resources can still not be used by human society because they are too small in physical scale, and it would be better for agents not to use them. Therefore, the tragedy of the anticommons can be summed up as the tragedy of over-fragmentation of resources *per se* or property rights, which leads to over-excludability and further leads to underuse of resources.

**FIGURE 1:**
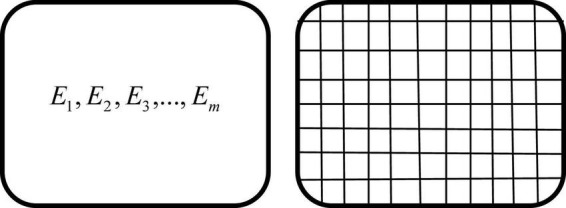
Legal anticommons and spatial anticommons.

For the sake of straightforwardness, a simple diagram can depict the difference between them both. The left side of Figure 1 represents the legal anticommons, which refers to the situation where resources are complete in physical space, but (*m*, *m*≫1) owners have excluding or even veto rights to resources. The right part illustrates the spatial anticommons, indicating that the resource is divided into too many components in physical space (*n*, *n*≫1), and the same person or organization owns no two adjacent fragmented resources at the same time. When the over-segmentation of physical space is caused by legal authorization, it is more reasonable to judge it as spatial anticommons rather than legal anticommons as long as each slice of resources after segmentation does not show excessive excludability. It is not difficult to imagine that for both the law type and the space type, stakeholders can usually find out ways to integrate resources to realize the optimal allocation of resources, as long as *m* or *n* is small enough. But if *m* or *n* is too large, and individuals’ cognitive models, mental accounts, and interests are various enough, then the integration process will be hard to enforce because the transaction cost involved is too high. Therefore, the tragedy of the anticommons is usually limited to the situation where the number of excluders is too large and it is difficult to reach a consensus, so that the integration of resources or their property rights becomes too costly to achieve a positive net income, which finally makes it difficult to make effective use of resources.

Empty storefronts in Moscow and biomedical research dilemma in America, though with disputes, can be used to illustrate the tragedy of the commons and the tragedy of the anticommons, respectively. In the 1990s, after the collapse of the Soviet Union, Yeltsin government accepted the “shock therapy,” a radical privatization, suggested by the World Bank. However, in quite a long time after privatization, a lot of Moscow storefronts remained empty, merchants and customers were forced to trade all kinds of daily necessities, even clothing, in kiosks, which was to the surprise of Russia’s then policy makers including Gaidar, for they believed that after the privatization reform the market mechanism would automatically start to work (Heller, 1998). This situation can be attributed to the legal anticommons: The radical “shock therapy” reform led to over-fragmentation of property rights of storefronts and the emergence of too many excluders who were in a chaos without a unified coordination mechanism.

Heller and his co-author Eisenberg argue that the developed market economies are also faced with serious anticommons dilemma, especially the spatial anticommons tragedies, though with disputes. They contend, since the 1970s and 1980s, the United States has introduced a large amount of private investment in biomedical research. To protect the rights of R&D investors, official organizations grant patents to these private companies (Heller and Eisenberg, 1998). Because biomedical and genetic research is so complex, and patent examination is not strict enough, a large number of fragmented biomedical patents have been granted. Genetic engineering, on its own, is like a giant jigsaw board game. Each tiny piece of gene patent information has almost no practical value by itself. Only when they are integrated in an orderly way to form a certain scale can useful information be obtained to decode human genes. On the purely technological level, the United States should have developed a flood of new drugs and treatments that could have saved a lot of lives with the research and development efforts. Unfortunately, the cost of acquiring enough valid biomedical or gene patents from too many private companies, and the transaction cost resulting from excluders’ opportunistic behavior, are so high that many new drugs and treatments are being nipped in the bud. Since the 1970s, spending on drug research and development in the United States has risen steadily, while practical inventions have declined. Millions of people continue to suffer from avoidable ailments (Heller and Eisenberg, 1998).

### Symmetry of tragedies of the commons and the anticommons by game theory

Heller (1998), the founder of the tragedy of the anticommons theory, said that “Antiommons property can be understood as the mirror image of commons property.” Heller (1998) also proposed in number 258 footnote “… one answer may lie in game theory-modeling of the anticommons, a direction for future research.” But setting up game theory model does not seem to be the professional skills of jurists, so the task is naturally handed over to economists. Followed by his suggestion, some scholars tried to prove the “symmetry” between these two tragedies from the perspective of mathematical models, among which, the first were Buchanan, a Nobel Prize winner in economics, and his colleague Yoon in their manuscript “*Symmetric Tragedies: Commons and Anticommons*” (Buchanan and Yoon, 2000). Buchanan and Yong’s manuscript was followed by Schulz, Parisi, and Depoorter’s manuscript titled “*Fragmentation in Property: Towards a General Model*” (Schulz et al., 2002), and later followed by a manuscript titled “*Duality in Property: Commons and Anticommons*” (Parisi et al., 2005). These scholars agree that the tragedy of the commons is symmetrical with the tragedy of the commons. But this is mainly because their research method is pure mathematics or game theory, rather than through experimental data.

Buchanan and Yoon (2000) formally responded to Heller’s proposal and it seems that they were the first influential scholars of doing so in the academic community. They took advantage of algebra and geometry knowledge to construct a concise and persuasive “commons–anticommons” model based on a series of assumptions, and tried to prove their symmetry. They began by introducing a parking lot – a large vacant lot near a rural area, where alternative parking could be found a mile away, whose economic value monotonously increases with the number of cars. The main assumptions of the model are as follows: For this resource, there are equal number of excluders and competitive users under commons condition. There is no cooperation or collusion among users or excluders, for both the commons and the anticommons games, respectively. The production function of the resource value is linear. The operation of the “open space” parking lot does not need any cost, that is to say, the cost of using it is zero.

With the help of Cournot duopoly, Buchanan and Yoon (2000) constructed an algebraic model and discussed the economic efficiency of the parking lot in the case of the commons and the anticommons. The production function of the economic value of the parking lot is *P* = *a*−*bQ*. In the context of the commons game, the production function is *max*_*Q*_1__ *PQ*_1_ = (*a*−*bQ*_1_−*bQ*_2_)*Q*_1_. As for the anticommons game, the production function is ∑*P*_*i*_ = *a*−*bQ*. According to their respective first-order condition, the total economic rents of the parking lot in the game of the commons and the game of the anticommons in the case of *n* participants are the same, that is, $TR\left(commons\right)=TR\left(anticommons\right)=\frac{na^{2}}{b{\left(n1\right)}^{2}}$. As the number of participants *n* approaches infinity, the economic rent of the parking lot approaches zero.

**FIGURE 2:**
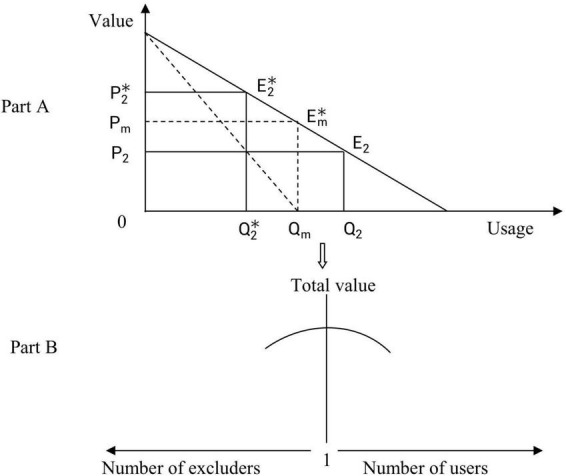
The symmetry between the commons and the anticommons.

To illustrate their models (especially the symmetry of the commons and the anticommons) more clearly and directly, Buchanan and Yoon (2000) also described the algebraic relations mentioned above from the perspective of geometric graphics, as shown in Figure 2. Obviously, when the right of usage and the right of excludability of the parking lot are given to just one person, the economic rent of the parking lot will be maximized: The maximum rent is $P_{m}0Q_{m}E_{m}^{*}$. When there are two people having excluding rights at the same time, the economic rent of the parking lot is $P_{2}^{*}0Q_{2}^{*}E_{2}^{*}$, which is of the same economic rent *P*_2_0*Q*_2_*E*_2_ when two people have the right to use the property. When the number of participants with usage rights or excluding rights becomes very large, the economic rent of the parking lot will be reduced to zero. Therefore, we can draw the following conclusion: Buchanan and Yoon (2000) established a mathematical model for the tragedy of the commons and the tragedy of the anticommons based on several hypotheses and proved the symmetry between them both from the perspective of pure mathematics. Their views have been followed and further studied by Schulz et al. (2002) and Ohkawa et al. (2012).

### Asymmetry of the commons and the anticommons from behavioral experiment perspective

It seems that Stewart and Bjornstad (2002) were the first scholars to verify the symmetry between the commons and the anticommons from the perspective of experimental economics. They published an online research report titled “*An Experimental Investigation of Predictions and Symmetries in the Tragedies of the Commons and Anticommons*,” attempting to provide an empirical basis for the mathematical model of the symmetry proposed by Buchanan and Yoon (2000). We guess their experiment design follows Ostrom et al. (1994). In one of their footnotes, they cited “Elinor Ostram, Roy Gardner, and James Walker, Rules, Games, and Common Pool Resources (Ann Arbor: University of Michigan Press, 1994),” but after prudent checks, we can confirm that the first author of this book is Elinor Ostrom, the first female winner of Nobel Prize in Economics, instead of Elinor Ostram. Their participants were recruited from undergraduate students taking introductory and advanced economics courses at the University of Tennessee. There were 278 participants in total. Participants were paid in cash, ranging from $9 to $22 per session which lasted for an hour and a half.

The experiment can be divided into two types: One was two participants in each group, while the other type was four participants in each group. From the viewpoint of the report itself, they claimed that they have verified Buchanan and Yoon’s (2000) symmetric model from the perspective of laboratory experiments. On the one hand, both types of experiments got symmetrical results of tragedies of the commons and the anticommons. On the other hand, to eliminate the interference caused by insufficient understanding of the experiment in previous rounds (a total of 14 rounds), they conducted a *t*-test by dropping the sample data of the first three rounds, and the result still stayed the same.

Later Stewart and Bjornstad (2002) were challenged and overturned by Vanneste, Hiel, Parisi, and Depoorter, a team consisting of economists, psychologists, and jurists. It seems that they do not object to the conclusion that the tragedy of the commons and the tragedy of the anticommons are symmetric from the perspective of game theory, partly because the third and fourth authors of Vanneste et al. (2006) are the first and third authors of Parisi et al. (2005), respectively, and Parisi et al. (2005) agree with the symmetry of the commons and the anticommons in the field of game theory. However, their experimental economics research results show that there is no symmetry between them – the tragedy of the anticommons is more serious than the tragedy of the commons. As their article titled “*From ‘Tragedy’ to ‘Disaster’: Welfare Effects of Commons and Anticommons Dilemmas”* shows, if the commons lead to tragedy, then the anticommons will lead to disaster (Vanneste et al., 2006).

They also designed two types of experiments; one in which participants were informed of game principles (collective rationality versus individual rationality) and the other type was the “uninformed” situation. Each experiment was designed as two different game categories – the commons game and the anticommons game. Both kinds of commons experiments, whether informed or uninformed, lab experiments or scenario experiments, proved that agents over-exploited the resource; Likewise, both kinds of anticommons experiments, whether informed or uninformed, lab experiments or scenario experiments, proved that the resource were under-exploited. In fact, these results had been proved for many times before them. Their marginal contribution is that Vanneste et al. (2006) prove that the welfare loss caused by the anticommons is severer than that caused by the commons, which is different from the symmetry conjecture by game theory, and it is also different from the report of Stewart and Bjornstad (2002).

Based on the following facts, the results of Vanneste et al. (2006) are much more credible. First, there are some spelling mistakes in the report of Stewart and Bjornstad (2002). For example, as mentioned above, they spelled Ostrom, the first woman who won the Nobel Prize in economics, as Ostram. Second, the link of their report seems invalid now, though it was available 3 years ago. Third, the article of Stewart and Bjornstad (2002) is just a report instead of a formally published journal manuscript. Forth, Vanneste et al. (2006) are an interdisciplinary team researching social dilemma issues, especially the commons and the anticommons. Thus it is relatively safe to conclude that the symmetry of the tragedy of the commons and the tragedy of the anticommons in the experimental field is overturned. Meanwhile, the conclusion that the anticommons tragedy is more serious than the commons tragedy in the experimental field is also recognized by Professor Heller, the founder of the anticommons theory.

The tragedy of the commons and the tragedy of the anticommons have been proved to be symmetrical in the game theory field. Still, their symmetry is finally refuted by experimental research, which is a paradox. We attempt to provide an in-depth analysis of this paradox from the cognitive psychology perspective.

## A brief review of cognitive psychology of economic behavior

This manuscript briefly reviews the evolution of cognitive psychology of economic behavior from perspectives of Simon’s bounded rationality, Kahneman and Tversky’s prospect theory, and Thaler’s mental accounting.

### Bounded rationality

At least since Aristotle in ancient Greece, thinkers have regarded human beings as advanced, rational animals except in a few extreme cases, such as drunkenness and rage. This thought, combined with the assumption of maximizing individual interests, forms the homo oeconomicus hypothesis in economics. As early as 1947, the great generalist Herbert Simon noticed the importance of psychological cognition on economic decisions and activities. His research shows that decision-makers cannot be completely rational, and what they can achieve is only limited rationality. In reality, decision-makers rely on experiences under the guidance of heuristics or the thumb rule and only follow the satisfaction principle (Simon, 2011). In addition, Simon pushed the study of behavioral economics to a new climax. In 1978, he won the Nobel Prize in economics for his pioneering research on the decision-making process of economic organizations. His theory of bounded rationality and heuristics were followed by many researchers, including Kahneman and Thaler, who also won the Nobel Prize in economics.

### Prospect theory

The prospect theory put forward by Kahneman and Tversky (1979) follows Simon’s bounded rationality hypothesis. They believe that people rely on finite heuristics to make decisions in an uncertain world. Prospect theory effectively combines psychological and economic research, reveals the decision-making mechanism under uncertainty, and opens up a new research field. The prospect theory is built based on expected utility theory which follows the expected value theory.

**FIGURE 3:**
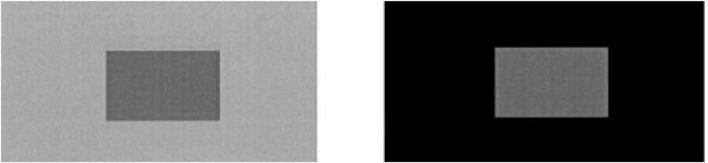
Visual renderings in different backgrounds.

According to the prospect theory, when making a decision, people choose a reference point and compare the alternatives with their reference point, which is often the *status quo*. The reference point effect is sometimes called the anchoring effect, similar to the visual background effect in Figure 3. For example, a plain loaf of bread significantly differs in satisfaction or utility for an individual in different situations. To the individual who has just had enough delicious and nutritious food, this plain loaf of bread is likely to be unattractive and with little utility or even negative utility. For the same individual who is now hungry and has no better choices, this plain loaf of bread is very satisfactory and has very high positive utility. In Figure 3, the situation of having had enough delicious food in advance is like the black background on the right side, while the situation of being very hungry and with no better choices is like the gray background on the left side. Under different backgrounds, the small rectangles with the same color in the center show obvious difference in visual perception: The small rectangle on the left seems much darker than that on the right.

Kahneman (2011) also follows the two-system brain model developed by psychologists Keith Stanovich and Richard West. In Kahneman’s view, system 1 exists like instinct, operating unconsciously and quickly, with little mental effort or sensation and complete autonomous control. System 2, however, shifts attention to mental tasks, such as complex calculations. The operation of system 2 is often associated with our subjective experiences of behavior, choice, and concentration. Kahneman points out that system 1 continuously provides system 2 with impressions, intuitions, intentions, and feelings. If system 2 receives this kind of information, it turns impressions, intuitions and so on into beliefs, and impulses into voluntary action. Normally, when everything is running smoothly, system 2 will directly accept suggestions from system 1. But when system 1 is blocked, system 2 is called for support, and then system 2 is activated. Kahneman’s thoughts can be found in his influential book titled “*Thinking, Fast and Slow*.” Kahneman won the Nobel Prize in economics in 2002 for his work in integrating psychology into economics, particularly how human decision-making impacts economic decisions in the situation of uncertainty. It is believed that Tversky, his collaborator, would have shared the prize with Kahneman had he not died in 1996.

In addition, according to the prospect theory, especially the value function, there are the following viewpoints: Individuals in the real world do not maximize utility but just react to possible or perceived changes in gains or losses, which are emotional and short-termed. Most individuals are risk-averse when receiving gains, and most individuals are risk-preferred when faced with losses. Generally speaking, individuals are more sensitive to losses than to gains.

Kahneman and Tversky’s prospect theory, especially the value function, greatly influences Thaler, their extraordinary follower and student, based on which his mental accounting theory was built. Meanwhile, the value function is also an important theoretical tool to analyze the paradox of the tragedy of the commons and the tragedy of the anticommons.

### Mental accounting

The main founder of the mental accounting theory is Thaler. Thaler (1999) defined accounting as “the system of recording and summarizing business and financial transactions in books, and analyzing, verifying, and reporting the results.” Tversky and Kahneman (1981) defined a mental account as: (i) the set of elementary outcomes that are evaluated jointly and how they are combined and (ii) a reference outcome that is considered neutral or normal. Thaler (1985) further defined mental accounting as the cognitive operations used by individuals and households to organize, evaluate, and keep track of financial activities. According to Thaler’s theory, no matter the gains or losses, in the actual world, people tend to treat them in different mental accounts even though they are of the same amount of money. At first, Thaler named it as psychological accounting, which Kahneman and Tversky, his teachers and collaborators, later changed as mental accounting. And Thaler finally adopted their suggested change.

According to traditional neoclassical economics, there is no difference between two sums of money of the same amount. Therefore, the principle of fungibility makes sense. But in Thaler’s opinion, even sums of money with equal purchasing power have different psychological value in the eyes of their owners. This kind of phenomenon is more obvious if it is personal belongings instead of money. The endowment effect can explain part of this. Thaler proposes endowment effect, which states that once people owns an item, they tend to overvalue it. In this way, Thaler changed the principle of fungibility followed by neoclassical economics into the principle of non-fungibility.

The cognitive theories of Kahneman, Tversky, Thaler, and others follow the underlying logic of Bentham’s pursuit of the greatest happiness. Thaler divides the utility people get from a transaction into two parts: Acquisition utility and transaction utility. Acquisition utility can be considered as consumer surplus of neoclassical economics, which measures the utility caused by the difference between consumer’s willingness to pay minus the actual market price of a good. While the transaction utility measures the utility resulting from the difference between a commodity’s actual price and its perceived reference price. In other words, transaction utility is like the psychological perception of whether and how good a deal is in an individual’s mind. If one thinks that the actual price is below the perceived reference price, he tends to believe that he made a good bargain. For example, if you ask a friend of yours to buy a sunhat, he spent 20 dollars in buying it. Suppose you trust all he said. If he tells you that he bought it from an upscale shopping center, you tend to think that you have got a lot of utility from this transaction, and the transaction utility is very high. While if your friend tells you he bought it from a grocery store downstairs that sells cheap commodities, then you are more likely to think it is not such a good deal and the transaction utility is low or even negative. Here is another example illustrating transaction utility. Some consumers cannot stop buying on-sale goods even though they do not need them at all. This is because, in their view, high transaction utility can be obtained through such transactions. Transaction utility is an important supplement to the consumer surplus of neoclassical economics. Although it seems irrational, it matters on people’s consumption decisions. For example, after winning the Nobel Prize, Thaler was asked how he would use his prize money, the then 72-year-old economist joked, “I will try to spend it as irrationally as possible!”

Moreover, Mr. Thaler also has insights into how people think about sunk costs through the mental accounting theory. Based on the homo economicus assumption, previous neoclassical economics suggests that people should not cry over spilled milk. But in reality, Thaler says, people often refuse to give up because giving up is like admitting defeat. Instead, in many cases, they tend to invest more on doomed failure projects to cover up their previous mistakes.

## Illustrating the paradox from cognitive psychology perspective

From the perspective of pure mathematical game theory, researchers assume that the economic man is one hundred percent rational and one hundred percent self-interested, based on which they can simplify economic analysis process to the problem of maximizing individual interests under certain constraints. Starting from this point, researchers will naturally conclude that there is symmetry between the tragedy of the commons and the tragedy of the anticommons. But in reality, in addition to purely rational factors, the irrational side of decision-makers will also substantially impact their decision-making process. One of the most compelling insights is from the field of cognitive psychology.

**FIGURE 4:**
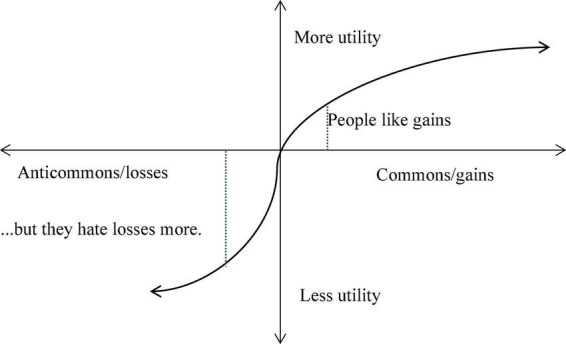
The valuation function of the commons vs. anticommons.

From the cognitive psychology perspective pioneered by Kahneman and Thaler et al., commoners in the commons situation are merely common users of their commonly owned resource. There is no clear definition of property rights toward the resource. That is, each commoner does not know which part of the resource belongs to him, so they have no rights to prevent others from using it. Therefore, in commoners’ psychological accounts, they tend to regard such resources as not “their own” in the first place, just commonly owned at best or even ownerless. Based on the knowledge of reference points and anchoring effect, what the commoners face is quite similar with the small central rectangle on the right side of Figure 3, with much lighter color by visual effect, i.e., they tend to think that their use rights are not that important. In terms of the value function, their use of common resources means gaining utility or value. In the situation of gaining utility, the increase of the utility function is relatively slow (the slope of the value function is small), as shown in Figure 4.

In the eyes of anti commoners, since each player has veto rights, others cannot use the resource without getting his permission. Thus they tend to mentally treat the resource as their own, and the relevant veto right seems like a precious privilege, even though other anti commoners have the same rights toward the resource. From anti commoners’ point of view, the veto right is similar with the small central rectangle on the left side of Figure 3, which seems darker than the one on the right side even though they are of the exact same degree of darkness in fact. Likewise, they think their right of excludability, especially the veto right under the anticommons game, is more important than the right of usage under the commons game. Therefore, in the eyes of anti commoners, to prevent others from encroaching on their “own cheese,” they tend to use their veto rights to prevent others from using the resource out of the consideration of risk aversion of loss, even if others pay them a price to buy their rights of excludability.

Affected by the endowment effect, anti commoners may overestimate their resource’s market value and the veto right. This is like a person selling his own house charging for $3 million, but if he is asked to buy the same house himself, his willingness to pay may be only $2.6 million, obviously less than his charging price as a seller. In the value function, the anticommons game is similar to people’s psychological response to loss. In the face of failure, the absolute value of negative utility generated by the loss of a sum of money is greater than the positive utility generated by the gain of the same amount of money. According to Thaler’s estimation, the pain brought by loss is about twice as much as the happiness brought by the gain (Thaler, 2015), as shown in Figure 4.

Our analysis can be further strengthened by the anchoring effect theory. When making a decision, the actor compares the changes resulting from the decision with a reference point (usually the *status quo*). Commoners tend to see their “status quo” as having no private property rights on the commonly owned resource. So they do not particularly care about their rights of usage. However, in the context of anticommons, anti commoners tend to regard anticommon resources as their own, so their reference point or “status quo” is as having got the valuable veto right, maybe a privilege in their eyes. To protect the resources and relevant veto rights from being diminished by others, they are more likely to conduct their veto rights than commoners to conduct their use rights. In both cases of commons and anticommons, players in both cases might get trading utility by conducting their relevant rights. But the trading utility of the anti commoners of excising their veto rights is presumably higher than that of the commoners exercising their use rights. Therefore, when the number of participants in both cases, and relevant resources are numerically or physically the same, the level of resource underuse caused by participants’ rights of excludability (manifested as veto rights) under the anticommons experiment, is greater than the level of overuse caused by common users under the commons experiment. This explains why according to pure game theory, the welfare loss caused by the tragedy of the commons (deadweight loss) and the tragedy of the anticommons should be equal, i.e., the two cases are symmetrical, but in the field of behavioral economics experiment, they are asymmetric, i.e., the deadweight loss caused by the tragedy of the anticommons is greater than that caused by the tragedy of the commons.

## Conclusion and future research direction

The paradox of the tragedy of the commons and the tragedy of the anticommons clearly shows that psychological cognition plays a tremendous and direct role in an individual’s decision-making process. We can even put it this way, psychological cognition determines an individual’s behavior. The brain of a real life decision-maker is not a cold, rational supercomputer, nor is its decision-making mechanism a set of computer programs. Although objective marginal revenue and marginal cost constitute the objective basis of the decision-making process, actor’s subjective selection, coding, and processing of these marginal costs and marginal revenue have a more direct influence on people’s decision-making process. Just like the neoclassical economics being criticized, cognitive psychology theories are still evolving or just changing, and there are some debates or disputes within cognitive psychology. For example, in the behavioral finance analysis by the prospect theory, Kahneman’s theoretical expectations vary greatly in specific scenarios. Whether the endowment effect holds water or not should also be further tested on a case-by-case basis. For example, a profound Chinese scholar and writer, Zhongshu Qian, who once worked as the vice-president of Chinese Academy of Social Sciences, in his world famous book titled “*Fortress Besieged*,” showed us that life is fortress besieged, i.e., people tend not to cherish what they have already had while be thirsty for what beyond their reach. Such viewpoints actually refute the endowment effect theory to some degree.

Although this manuscript discusses the symmetry of the commons and the tragedy of the anticommons from the perspective of cognitive psychology, it does not mean that deadweight losses caused by the commons dilemma and the anticommons dilemma in the real world are of the same level. The study of the symmetry of the tragedy of the commons and the tragedy of the anticommons is much more complicated if it is to appraise their real-life effects. The tragedy of the anticommons lies mainly in the economic welfare loss caused by participants’ underuse. Still, the resources *per se* will not be diminished or even totally destroyed due to their underuse behavior. However, overuse in the tragedy of the commons may result in deterioration or even total ruin of the resources *per se*. Therefore, prior researches on the symmetry between the tragedy of the commons and the tragedy of the anticommons, are only short-term analysis. If longer term future is taken into account, it is highly likely that the economic welfare loss caused by the tragedy of the commons is much higher than that caused by the tragedy of the anticommons. Meanwhile, apart from economic welfare, other factors such as social effect, ecological effect, equality and justice, and so forth, are also worth examining. Therefore, the paradox between the tragedy of the commons and the tragedy of the anticommons is still worth further discussion in the future. Based on our own research and prior literature, we suggest that future researches should focus more on real-world natural experiments and case-by-case studies with interdisciplinary efforts.

## Data availability statement

The original contributions presented in this study are included in the article/supplementary material, further inquiries can be directed to the corresponding authors.

## Author contributions

XY designed the research and wrote the main part of the manuscript. YW and SL wrote the section “Conclusion and future research direction,” offered modification suggestions and helped translate the manuscript. SY and SZ collected relevant literature and wrote part of the literature review section. All authors contributed to the article and approved the submitted version.
